# Altered Immune Landscape and Disrupted Coral-*Symbiodinium* Symbiosis in the Scleractinian Coral *Pocillopora damicornis* by *Vibrio coralliilyticus* Challenge

**DOI:** 10.3389/fphys.2019.00366

**Published:** 2019-04-02

**Authors:** Zhi Zhou, Shuimiao Zhao, Jia Tang, Zhaoqun Liu, Yibo Wu, Yan Wang, Senjie Lin

**Affiliations:** ^1^Key Laboratory of Tropical Biological Resources of Ministry of Education, Hainan University, Haikou, China; ^2^Liaoning Key Laboratory of Marine Animal Immunology and Disease Control, Dalian Ocean University, Dalian, China; ^3^Department of Marine Sciences, University of Connecticut, Groton, CT, United States

**Keywords:** coral bleaching, pathogen, immunol response, symbiosis, scleractinian coral

## Abstract

*Vibrio coralliilyticus* is known to cause coral diseases, especially under environmental perturbation, but its impact on coral physiology and underpinning mechanism is poorly understood. In the present study, we investigated cytological, immunological, and metatranscriptomic responses of the scleractinian coral *Pocillopora damicornis* to *V. coralliilyticus* infection. The density and chlorophyll content of symbiotic zooxanthellae decreased significantly at 12 and 24 h after *Vibrio* challenge. The activities of antioxidant enzymes such as superoxide dismutase and catalase, nitric oxide synthase, phenoloxidase (PO), and the activation level of caspase3 all rose significantly in *P. damicornis* after *Vibrio* challenge. In the metatranscriptomic analysis, we found 10 significantly upregulated genes in the symbionts at 24 h after the challenge, which were mostly involved in the metabolism of nucleic acid and polysaccharide, and 133 significantly down-regulated symbiont genes, which were mainly related to amino acid catabolism and transport. Meanwhile, 1432 significantly upregulated coral genes were revealed, highly overrepresented in GO terms that are mostly related to the regulation of immune response, the regulation of cytokine production, and innate immune response. Furthermore, at 24 h after *Vibrio* challenge, 890 coral genes were significantly downregulated, highly overrepresented in four GO terms implicated in defense response. These results in concert suggest that *V. coralliilyticus* infection triggered the innate immune response including the redox, PO, and apoptosis systems, but repressed the response of the complement system in the scleractinian coral *P. damicornis*, accompanied by symbiont density decrease and symbiosis collapse through disordering the metabolism of the symbionts. These findings shed light on the molecular regulatory processes underlying bleaching and degradation of *P. damicornis* resulting from the infection of *V. coralliilyticus*.

## Introduction

Reef-building scleractinian corals are the pillar of the biologically and economically important coral reef ecosystems. Scleractinian corals flourish in shallow tropical waters with low and often undetectable concentrations of dissolved inorganic nutrients, depending on the elaborate coral-*Symbiodinium* endosymbiosis (Fabina et al., 2013). The unicellular symbiont lives in the intracellular vacuoles of host gastrodermal cells, and supplies the host coral with indispensable organic nutrients and oxygen through photosynthesis (Loram et al., 2007). In return the host coral provides inorganic nutrients and carbon dioxide for symbionts. However, due to environmental changes and anthropogenic perturbations, scleractinian corals have been subjected to impacts of frequent and severe diseases over the past decades (Rosenberg and Ben-Haim, 2002).

Bacterial pathogens are one of main etiologies of scleractinian coral diseases, and pathogenic *Vibrio* has been found in several scleractinian corals. For example, *V. alginolyticus* has been identified to be the pathogenic agent of *Montastraea* spp. yellow band disease in Caribbean and Indo-Pacific and *Porites andrewsi* white syndrome in the South China Sea (Cervino et al., 2008; Zhenyu et al., 2013). *V. coralliilyticus* is a coral bleaching pathogen, which was characterized firstly as temperature-dependent pathogen (strain YB1) of scleractinian coral *Pocillopora damicornis* (Ben-Haim et al., 2003a), and therefore has received attention. It has been reported that *V. coralliilyticus* used dimethylsulfoniopropionate as a chemotaxis and chemokinesis to target the mucus of the coral host (Garren et al., 2014), and resulted in coral tissue lysis and symbiont density decrease through extracellular proteases (Ben-Haim et al., 2003b). Furthermore, it has been observed recently that non-virulent *V. coralliilyticus* induced an “efficient” immune response, whereas virulent one caused immuno-suppression in the coral host (Vidal-Dupiol et al., 2014). However, the molecular mechanisms underlying the pathogenesis process of *V. coralliilyticus* to scleractinian corals, especially for symbiosis collapse, remain relatively poorly understood and underexplored particularly by high-throughput transcriptome sequencing technology.

Scleractinian corals possess a suite of innate immune defense mechanisms that enable them to maintain a close association with bacterial communities and eliminate potentially harmful microbes (van de Water et al., 2015). Pattern recognition receptors such as lectin, Toll/NOD-like receptor, and scavenger receptor identified in scleractinian corals can recognize the potential bacterial pathogen (Kvennefors et al., 2008; Hamada et al., 2013; Neubauer et al., 2016). The components of Toll-like receptor-to-NF-κB signaling pathway have been identified, and their immune-related functions have also been confirmed (Libro et al., 2013; Poole and Weis, 2014; Williams et al., 2018). The redox system, phenoloxidase (PO) system, complement system, antimicrobial peptide, apoptosis, and autophagy of scleractinian coral all have been thought to be involved in the killing and elimination of pathogenic microbes (Palmer et al., 2011; Vidal-Dupiol et al., 2011a; Brown et al., 2013; Fuess et al., 2017). Furthermore, innate immunity in scleractinian corals is implicated not only in the defense against invasive pathogenic microbes but also in the maintenance of coral-*Symbiodinium* symbiosis (Kvennefors et al., 2010; Zhou et al., 2017).

*Pocillopora damicornis* is a scleractinian coral in the family Pocilloporidae, and is widely distributed in the tropical and subtropical regions of the Indian and Pacific Oceans. It has been used as a model to address effects of *Vibrio* infection (Vidal-Dupiol et al., 2011b) and interactive effects of thermal stress and *Vibrio* infection (Garren et al., 2014). To elaborate the immune defense mechanism of scleractinian coral to pathogenic *Vibrio* as well as its effect on coral-*Symbiodinium* symbiosis, the symbiont density and chlorophyll content, the crucial immune parameters, and the metatranscriptome of coral *P. damicornis* were investigated after the challenge of pathogen *V. coralliilyticus*. The results provide insights for further understanding the potent functions and potential mechanisms of innate immune response in pathogen elimination and symbiosis maintenance in the scleractinian coral.

## Materials and Methods

### Coral

Colonies of the scleractinian coral *P. damicornis* were collected from a coral reef in Wenchang, Hainan Province, China, and transported to Hainan University where it was cultured in flow-through aquaria (ca. 500 L) filled with natural seawater (26°C). Cultures were illuminated with LED light bulbs in a 12 h/12 h light–dark cycle (light intensity 518 μmol photons m^-2^ s^-1^) for 1 month to acclimatize in laboratory conditions.

### *Vibrio* Challenge Experiment

The Gram-negative bacterium *Vibrio coralliilyticus* SCSIO 43001 was kindly provided by Dr. Xiaoxue Wang and grown in marine broth 2216E at 28°C. Live *V. coralliilyticus* were added into the natural seawater with a final concentration of 10^8^ CFU ml^-1^, and meanwhile a total of 60 coral nubbins were prepared for the *Vibrio* challenge experiment. Then, 25 of the coral nubbins were transferred into the *Vibrio*-containing seawater, hereafter referred to as the *Vibrio* challenge group, while another 25 were used as the control group, which were incubated only in the natural seawater, and the remaining 10 were the blank group. Coral nubbins were randomly sampled in the *Vibrio* challenge and control groups after 6, 12, and 24 h incubation, while the blank group was randomly sampled at 0 h. The three nubbins from each of the *Vibrio* challenge and control groups after 24 h exposure were immediately stored in liquid nitrogen for subsequent RNA extraction and transcriptome sequencing. Meanwhile, six nubbins were prepared from each group at each time point and used for evaluating the density and chlorophyll concentration of symbiotic zooxanthellae, and crucial immune parameters of the coral host.

### The Density Determination of Symbiotic Zooxanthellae

The variation of symbiont density after *Vibrio* challenge was determined by the method described by Higuchi et al. (2008, 2015) with few modifications. Briefly, tissue homogenates were prepared using Waterpik water jet to strip the tissue from the coral skeleton into approximately 10 ml filtered seawater. Two milliliter homogenates were then centrifuged at 5000 rpm, 4°C for 15 min, and the supernatants were used to determine coral enzyme activities. The symbionts were resuspended in filtered seawater, and counted using a Neubauer hemocytometer (QIUJING, China). The surface area of the nubbins was determined by the aluminum foil method (Johannes et al., 1970). The density of symbiotic zooxanthellae was defined as the ratio of the symbiont number to the surface area of coral nubbin (cell cm^-2^).

### The Measurement of Chlorophyll Content

The chlorophyll content of symbiotic zooxanthellae was determined after *Vibrio* challenge using the method described by Hedouin et al. (2016). Briefly, 2 ml homogenates were centrifuged at 5000 rpm, 4°C for 10 min to obtain symbionts. The harvested symbionts were resuspended in filtered seawater, and then centrifuged at 12,000 rpm, 4°C for 30 s. Chlorophyll *a* and *c* were extracted for 24 h at 4°C in 100% acetone, following by a centrifugation at 12,000 rpm, 4°C for 30 s. The absorbance values of the extracts were measured at 630 and 663 nm. The total content of chlorophyll *a+c_2_* was computed according to the equations of Jeffrey and Humphrey (1975). The chlorophyll content was the ratio of the total content of chlorophyll *a+c_2_* to the number of symbionts, and expressed as pg cell^-1^.

### Activity Assay of Antioxidases and Nitric Oxide Synthetase

Total activities of superoxide dismutase (SOD), catalase (CAT), and nitric oxide synthetase (NOS) in the supernatants were measured using commercial kits (JIANCHENG, A001, A007, and A014), following the manufacturer’s recommendations. Total SOD activity was determined by the hydroxylamine method, where 1 SOD activity unit was defined as the enzyme amount causing 50% inhibition in 1 ml reaction solution. Total CAT activity was determined using spectrophotometry to measure the yellowish complex compound generated after the reaction between hydrogen peroxide and ammonium molybdate. Here, 1 CAT activity unit referred to the amount of enzyme needed to degrade 1 mmol hydrogen peroxide per second. Spectrophotometry was also used to determine total NOS activity by measuring the production of NO synthesized by NOS using L-arginine as the substrate. For this, 1 NOS activity unit represented the amount of enzyme catalyzing the production of 1 nmol NO per min. After the concentration of total protein in the supernatant was quantified by BCA method (Dodd and Drickamer, 2001), the measured enzyme activity was divided by the total protein to yield specific activity expressed as U mg^-1^ protein.

### Activity Assay of Phenoloxidase (PO)

Phenoloxidase activity was measured by the photocolorimetric method described by Palmer et al. (2011) with slight modifications. L-DOPA (Sigma-Aldrich) was used as substrates, and the formation of *o*-quinones was recorded spectrophotometrically. Briefly, 20 μl supernatant was mixed with 65 μl PBS in the well of microplate (Costar). After the addition of 30 μl of 10 mmol l^-1^ L-DOPA (in PBS), the absorbance at 490 nm in each reaction well was measured every 5 min for 45 min using a microplate spectrophotometer (TECAN, infinite F50). The PO activity was defined as the change of absorbance (∆OD_490_) catalyzed by 1 mg supernatant protein in 1 min for the linear portion of the reaction curve, and expressed as ∆OD_490_ mg^-1^ min^-1^.

### Caspase3 Assay

The caspase3 activity in the supernatant was measured by Caspase-3 Colorimetric Assay Kit (KeyGEN BioTECH) according to the instruction. Briefly, the supernatants of all samples were diluted firstly to the same protein concentration. Then, 50 μl supernatants were added in the reaction mixture containing 50 μl reaction buffer and 5 μl substrate. After an incubation in the dark at 37°C for 4 h, the color change was detected spectrophotometrically at the wavelength of 405 nm. The activity of caspase3 was defined as the absorbance of the reaction solution at 405 nm (OD_405_), and the activation level of caspase3 in the scleractinian coral was defined as the ratio of OD_405_ in samples to that of the blank group.

### Deep Sequencing of *P. damicornis* Metatranscriptome

The coral nubbins with symbionts were ground in liquid nitrogen and mixed with 1 ml Trizol reagent (Invitrogen). The mixture was transferred into a 1.5 ml centrifuge tube and centrifuged at 5000 rpm, 4°C for 5 min to discard calcium carbonate skeleton. Subsequently, total RNA was isolated according to the protocol described by Stefanik et al. (2013). The extracted total RNA was quantified by Nanodrop 2000 (Thermo Scientific) at 260/280 nm (ratio > 2.0) and its integrity was checked with Angilent 2100 Bioanalyzer (Agilent Technologies). After the total RNA extraction and DNase I treatment, magnetic beads with Oligo(dT) are used to isolate mRNA. Then, the paired-end fragment library (2 × 150 bp) was constructed and sequenced on the Illumina HiseqX platform according to the manufacturer’s instructions (BGI, Shenzhen, China). The generated raw sequencing reads were deposited at the NCBI Short Read Archive (SRA) under Accession No. SRP133521.

### Reads Mapping and Identification of Differentially Expressed Genes

Raw reads obtained from Illumina sequencing were processed by the Fastx-toolkit pipeline^1^ to summarize data production, evaluate sequencing quality, and remove low quality reads and adaptor sequences. Quality filtration removed sequences in which 80% of the base pairs had a Phred score of less than 20, while adaptor sequences were removed using the trimmer package. The assembled coral and *Symbiodinium* transcripts in our previous study served as reference sequences for reads mapping, and the mapping of paired-end reads was performed using the HISAT2 software (Pertea et al., 2016). After reads mapping, the resultant BAM files were used by StringTie and DESeq2 software to estimate transcript abundances and identify differentially expressed genes between the *Vibrio* challenge and control groups.

### GO Enrichment of Differentially Expressed Genes

After identification of differentially expressed genes, the lists of significantly upregulated and downregulated genes were generated for both the scleractinian coral and symbionts. GO enrichment analysis was implemented via the hypergeometric test with filter value of 0.01. The significantly upregulated or downregulated genes were selected as test set, while all assembled coral or symbiont genes were used as the reference set. The BiNGO tool was employed to calculate the overrepresented GO terms in the network and display them as a network of significant GO terms.

### Statistical Analysis

All data were presented as means ± standard deviation (SD). All of the data were subjected to two-way analysis of variance (two-way ANOVA) followed by multiple comparison (SNK) to determine significant differences among the treatments and controls. Differences were considered significant at *p* < 0.05.

## Results

### Density and Chlorophyll Content of Symbiotic Zooxanthellae After the Challenge of *V. coralliilyticus*

**FIGURE 1:**
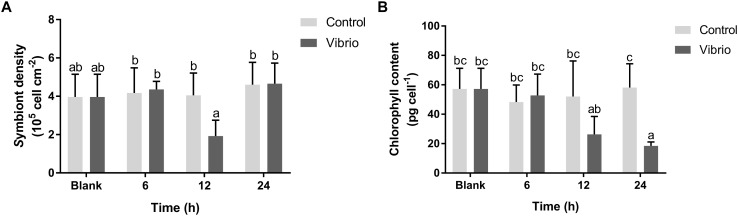
Density **(A)** and chlorophyll **(B)** variation of the symbionts in the scleractinian coral *Pocillopora damicornis* after *Vibrio* challenge. Vertical bars represent the mean ± SD (*N* = 6), bars with different letters were significantly different (*p* < 0.05).

The density of symbiotic zooxanthellae and their chlorophyll content both decreased significantly in *P. damicornis* after *Vibrio* challenge. The symbiont density in the *Vibrio* challenge group was significantly lower (1.92 ± 0.83 × 10^5^ cell cm^-2^, *p* < 0.05) after 12 h of pathogen exposure, in comparison with those in the blank (3.96 ± 1.19 × 10^5^ cell cm^-2^) and control groups (4.05 ± 1.16 × 10^5^ cell cm^-2^) (Figure 1A). The chlorophyll content of symbiotic zooxanthellae began to decrease at 12 h, and reached the lowest level (18.43 ± 2.71 pg cell^-1^, *p* < 0.05) at 24 h after *Vibrio* challenge (Figure 1B). There was no significant difference in both symbiont density and chlorophyll content between the control and blank groups for the entire duration of the experiment.

### Effects of *Vibrio* Challenge on the SOD, CAT, and NOS Activities

**FIGURE 2:**
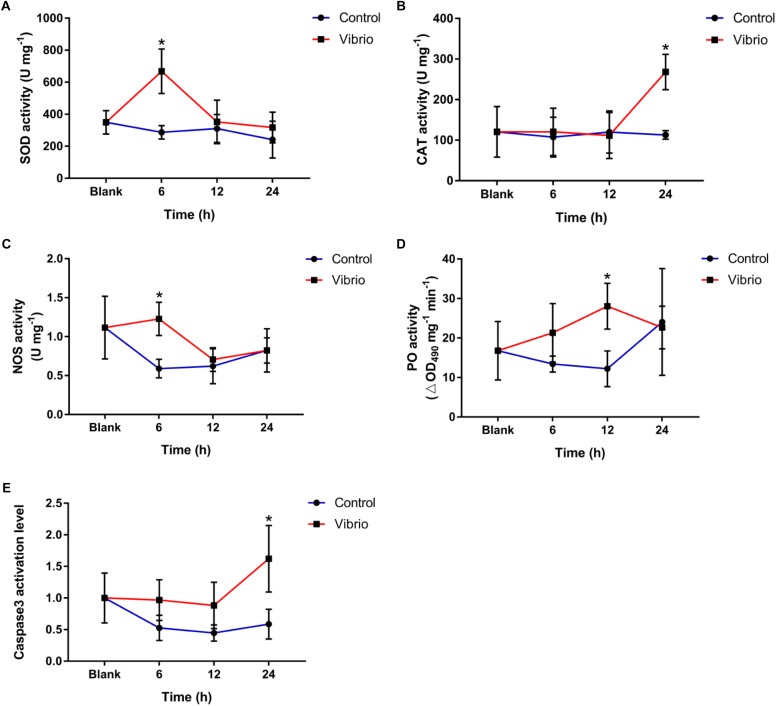
Temporal changes of superoxide dismutase (SOD, **A**), catalase (CAT, **B**), nitric oxide synthetase (NOS, **C**), and phenoloxidase (PO, **D**) and caspase3 activation level **(E)** in the scleractinian coral *Pocillopora damicornis* after *Vibrio* challenge. Vertical bars represent the mean ± SD (*N* = 6), and bars with asterisks are significantly different (*p* < 0.05).

The activities of SOD, CAT, and NOS increased significantly in *P. damicornis* after *Vibrio* challenge. For SOD activity, the significant increase only occurred at 6 h (668.20 ± 138.97 U mg^-1^, *p* < 0.05) after *Vibrio* challenge (Figure 2A). CAT activity increased significantly (268.09 ± 43.58 U mg^-1^, *p* < 0.05) at 24 h after *Vibrio* challenge, which was significantly higher than those in the blank and control groups (Figure 2B). The NOS activities at 6 h (1.23 ± 0.21 U mg^-1^, *p* < 0.05) after *Vibrio* challenge were higher than those in the control group (Figure 2C). There was no significant difference in the activities of SOD, CAT, and NOS between the control and blank groups for the entire duration of the experiment.

### Effect of *Vibrio* Challenge on the PO Activities and Caspase3 Activation Levels

The PO activity reached the peak level at 12 h of *Vibrio* challenge (28.05 ± 5.80 ∆OD_490_ mg^-1^ min^-1^, *p* < 0.05), which was significantly higher than that in the control group (Figure 2D), while no significant difference was observed at the other time points. The caspase3 activation level increased significantly in the scleractinian coral *P. damicornis* after *Vibrio* challenge, and it reached the peak (1.62 ± 0.53-fold, *p* < 0.05) at 24 h after *Vibrio* challenge (Figure 2E). There was no significant difference in the caspase3 activation level between the control and blank groups for the entire duration of the experiment.

### Construction and Sequencing of Transcriptome Libraries

A total of six paired-end transcriptome libraries were constructed, including three libraries in the control group and three libraries in the *Vibrio* challenge group. To compare the abundance and difference of gene expression, these six libraries were sequenced to saturated level. After filtration of low-quality sequence and adaptor sequence, a total of 224,425,705 paired-end reads with a length of 2 × 150 bp were obtained, including 38,941,120, 40,536,014, and 41,786,953 reads from three control libraries, and 36,306,952, 33,218,877, and 33,635,789 reads from three *Vibrio* challenge libraries, respectively (Table 1).

**Table 1:** Transcriptome mapping statistics.

Library	Total reads	Mapped rate to coral transcripts (%)	Mapped rate to zooxanthella transcripts (%)
Control_1	38,941,120	46.50	34.25
Control_2	40,536,014	42.46	37.50
Control_3	41,786,953	39.44	42.18
Vibrio_1	36,306,952	40.32	40.28
Vibrio_2	33,218,877	25.29	56.34
Vibrio_3	33,635,789	49.04	30.25


### Gene Expression

The high-quality clean paired-end reads obtained from all six transcriptome libraries were processed in HISAT2 software to map to the reference sequences built from the assembled coral and *Symbiodinium* transcripts in our previous study. The mapping rates of the six libraries ranged from 25.29 to 49.04% for coral and from 30.25 to 56.34% for zooxanthella (Table 1). The gene expression level was analyzed using StringTie software, and the counts of mapped reads for each coral and symbiont gene were further fed into the DESeq2 software to identify differentially expressed genes (Supplementary Tables S1, S2). Out of the 38,951 genomically predicted coral genes, 36,534 and 36,306 genes (more than three reads) were detectable in the control and *Vibrio* challenge groups, respectively. Meanwhile, we detected 45,357 and 45,207 genes of the genomically predicted 489,305 symbiont genes in the control and *Vibrio* challenge groups, respectively.

After library calibration, the expression levels of these genes were compared between the control and *Vibrio* challenge groups. The result revealed 2322 differentially expressed coral and only 143 zooxanthella genes (Table 2), which accounted for 5.96 and 0.03% of total number of coral and zooxanthella genes, respectively. There were 1432 significantly upregulated and 890 significantly downregulated genes for the scleractinian coral, while 10 and 133 for the symbionts (Supplementary Tables S3, S4).

**Table 2:** The number of differentially expressed genes after *Vibrio* challenge.

	Coral	Zooxanthella
Total assembled genes	38,951	48,935
Differentially expressed genes	2322	143
Significantly upregulated genes	1432	10
Significantly downregulated genes	890	133


### Functional Annotation of the Differentially Expressed Genes in the Symbiotic Zooxanthellae

Ten significantly upregulated genes and 133 significantly downregulated genes were observed in the symbiont at 24 h after *Vibrio* challenge. Blastx searches revealed these significantly upregulated genes including four hypothetical protein genes, one hypoxia-inducible factor 1-alpha inhibitor, one endoglycoceramidase, one glycine–tRNA ligase, one DEAD-box ATP-dependent RNA helicase, one DNA ligase, and one putative transport protein (Table 3).

**Table 3:** The significantly upregulated genes in the *Symbiodinium* after *Vibrio* challenge.

	Gene ID	Length (bp)	Blastx_best_hit
1.	TRINITY_DN51156_c1_g2	1024	Hypothetical protein AK812_SmicGene16484 [*Symbiodinium microadriaticum*]
2.	TRINITY_DN23602_c0_g2	1224	Hypothetical protein AK812_SmicGene23094 [*Symbiodinium microadriaticum*]
3.	TRINITY_DN49544_c1_g1	1452	Hypothetical protein AK812_SmicGene15080 [*Symbiodinium microadriaticum*]
4.	TRINITY_DN34152_c2_g1	1463	Hypothetical protein AK812_SmicGene12542 [*Symbiodinium microadriaticum*]
5.	TRINITY_DN37729_c0_g2	266	Hypoxia-inducible factor 1-alpha inhibitor [*Symbiodinium microadriaticum*]
6.	TRINITY_DN47979_c0_g3	2121	Endoglycoceramidase [*Symbiodinium microadriaticum*]
7.	TRINITY_DN38478_c0_g4	749	Glycine-tRNA ligase [*Symbiodinium microadriaticum*]
8.	TRINITY_DN43833_c2_g7	998	DEAD-box ATP-dependent RNA helicase 40 [*Symbiodinium microadriaticum*]
9.	TRINITY_DN36679_c4_g2	2799	DNA ligase [*Symbiodinium microadriaticum*]
10.	TRINITY_DN37519_c3_g1	1177	Putative transport protein [*Symbiodinium microadriaticum*]


**FIGURE 3:**
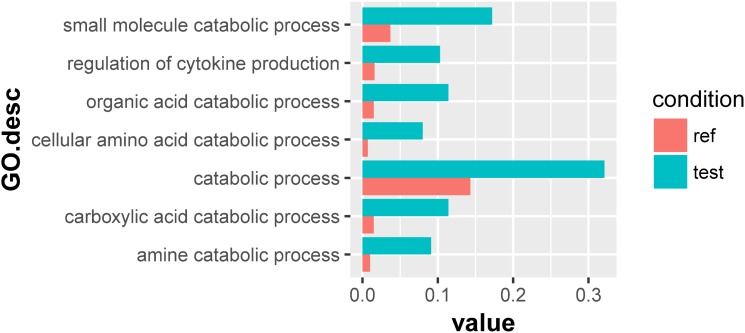
Overrepresented GO terms of the 133 significantly downregulated genes in the symbionts of the scleractinian coral *P. damicornis* at 24 h after *Vibrio* challenge. A total of seven GO terms were overrepresented for these significantly downregulated genes. “Ref” referred to the proportion of genes annotated to a GO term in all reference gene of symbiotic zooxanthellae, while “test” referred to the proportion of genes annotated to that GO term in all significantly downregulated genes.

For the 133 significantly downregulated genes, their GO overrepresentation was analyzed at multiple GO levels in the Biological Process category. A total of seven major GO terms were overrepresented, which were mainly related to the regulation of cytokine production and the catabolic processes of small molecules, organic acids, cellular amino acids, carboxylic acid, and amine (Figure 3 and Supplementary Table S5). Furthermore, three transporter-related genes were observed through Blastx searches, including intraflagellar transport protein (TRINITY_DN54551_c5_g3), sodium-coupled neutral amino acid transporter (TRINITY_DN48832_c0_g1), and sodium-dependent dicarboxylate transporter (TRINITY_DN51317_c0_g2) (Supplementary Table S6).

### Functional Annotation of the Differentially Expressed Genes in the Scleractinian Coral

The expression levels of 1432 coral genes in the *Vibrio* challenge group were higher than those in the control group, while the expression levels of 890 genes were significantly lower. The GO overrepresentation of these differentially expressed genes was further analyzed at multiple GO levels in the Biological Process category.

**FIGURE 4:**
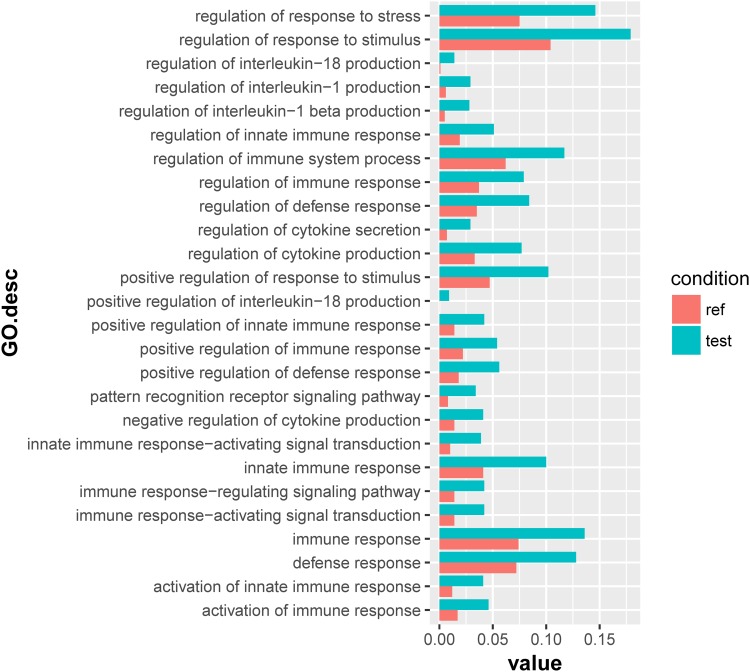
Overrepresented GO terms of the 1432 significantly upregulated genes in the scleractinian coral *P. damicornis* at 24 h after *Vibrio* challenge. A total of 26 GO terms were overrepresented for these significantly upregulated genes. “Ref” referred to the proportion of genes annotated to a GO term in all reference gene of the scleractinian coral *P. damicornis*, while “test” referred to the proportion of genes annotated to that GO term in all significantly upregulated genes.

**FIGURE 5:**
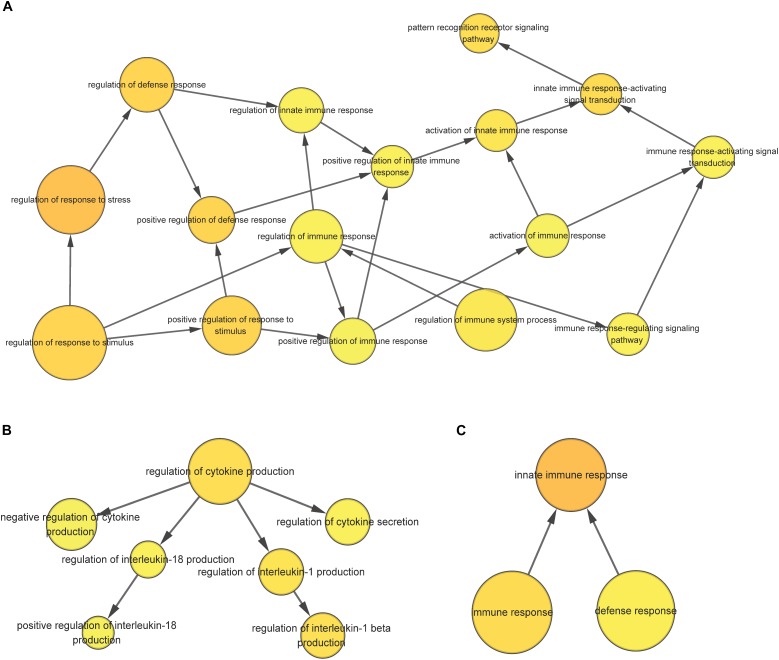
The overrepresented GO term group correlated with the regulation of immune response **(A)**, the regulation of cytokine production **(B)**, and innate immune response **(C)** for significantly upregulated genes in the scleractinian coral *P. damicornis* at 24 h after *Vibrio* challenge.

**FIGURE 6:**
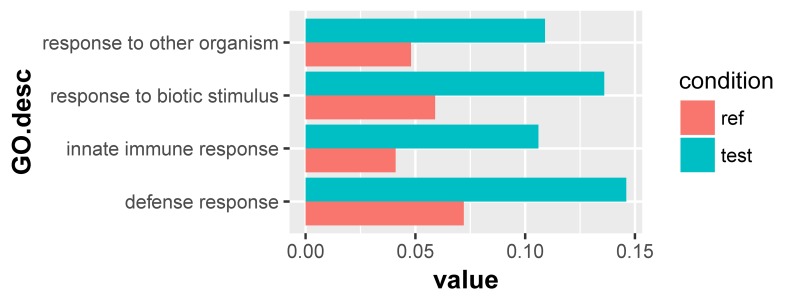
Overrepresented GO terms of the 890 significantly downregulated genes in the scleractinian coral *P. damicornis* at 24 h after *Vibrio* challenge. A total of four GO terms were overrepresented for these significantly downregulated genes. “Ref” referred to the proportion of genes annotated to a GO term in all reference gene of the scleractinian coral *P. damicornis*, while “test” referred to the proportion of genes annotated to that GO term in all significantly downregulated genes.

For the 1432 significantly upregulated genes, there were 26 major overrepresented GO terms (Figure 4 and Supplementary Table S7). These overrepresented GO terms could be divided into three groups, including the regulation of immune response, the regulation of cytokine production, and innate immune response (Figure 5). The first group contained the activation and positive regulation of immune response, and immune signaling pathway. Furthermore, a total of four overrepresented GO terms were observed for the 890 significantly downregulated genes (Figure 6 and Supplementary Table S8), which were mainly related to defense response. Of these two GO terms were overrepresented for both significantly upregulated genes and significantly downregulated genes, including GO:0045087 *innate immune response* and GO:0006952 *defense response*.

### Functional Annotation of the Differentially Expressed Genes Under Common Overrepresented GO Terms

Differentially expressed genes contained in two common overrepresented GO terms (GO:0045087, GO:0006952) mentioned above were annotated through NCBI blastx, and the genes under GO:0045087 was a part of those under GO:0006952 (Supplementary Tables S9–S12). For GO:0006952, more upregulated genes were related to positive regulation of immune response than downregulated genes, such as macrophage mannose receptor, Toll-like receptor, NACHT, LRR, and PYD domains-containing protein and TNF receptor-associated factor. Furthermore, the genes of complement components, including complement C3 and CUB, were exclusively downregulated genes (Table 4).

**Table 4:** The number of crucial immune genes significantly upregulated and downregulated in *P. damicornis* at 24 h after *Vibrio* challenge.

Gene	Upregulation	Downregulation
Lectin	5	1
NACHT, LRR, and PYD domains-containing protein	13	5
TNF receptor-associated factor	6	1
E3 ubiquitin-protein ligase	3	5
Complement C3	0	2
CUB	0	1


## Discussion

The survival and reproduction of scleractinian coral is increasingly threatened by a wide variety of environmental insults, such as increased seawater temperature, ocean acidification, and pathogenic microorganisms. The Gram-negative bacterium *V. coralliilyticus* has been identified as a coral bleaching pathogen in the scleractinian coral *P. damicornis* and pathogenic agent of other corals; however, its molecular mechanism underlying the effect on coral physiology is still not well understood (Ben-Haim et al., 2003a,b; Kimes et al., 2012). Through physiological, biochemical, and transcriptomic approaches, we have investigated effects of *V. coralliilyticus* challenge on *P. damicornis* and its symbiotic zooxanthellae and found some interesting responses.

### The Disruption of the Coral-*Symbiodinium* Symbiosis After *Vibrio* Challenge

*Vibrio coralliilyticus* SCSIO 43001 challenge led to significant decreases in the density and chlorophyll content of symbiotic zooxanthellae in *P. damicornis*, demonstrating that the symbiosis could be disrupted by *V. coralliilyticus* infection. This is consistent with a previous report that the infection of *V. coralliilyticus* YB1 caused the decline of density (cell g^-1^ of tissue) and chlorophyll content (chlorophyll *a*, μg g^-1^ of tissue) of symbionts in *P. damicornis*, eventually leading to coral bleaching and lysis (Ben-Haim et al., 2003b). The similar results suggest that the challenge of *V. coralliilyticus* SCSIO 43001 could also potentially result in symbiosis collapse and coral bleaching through depressing the symbiont density and chlorophyll content. The innate immune response of *P. damicornis* could be triggered by the infection with pathogen *V. coralliilyticus*, as previously shown to manifest in the induced significant change of the expression level of immune-related coral genes such as lectin, metal-binding protein, and cysteine protease inhibitor (Vidal-Dupiol et al., 2011b). Both the innate immune response and symbiosis collapse in *P. damicornis* as a result of *Vibrio* challenge suggest a cascade of pathogenic effects from eliciting immune response, decreasing symbiont abundance, and photosynthetic capacity (chlorophyll content), to causing coral bleaching or even degradation. Whether *V. coralliilyticus* can infect both the coral and *Symbiodinium* or the effect on *Symbiodinium* was a secondary impact stemming from the perturbed coral host remains to be further studied in the future.

### Immune Response of the Scleractinian Coral to *Vibrio* Challenge at the Biochemical Level

Scleractinian corals employ the innate immune response as the sole defense mechanism against pathogen infection, such as the redox, PO, and apoptosis systems (Palmer et al., 2010, 2011; Fuess et al., 2017). To understand the immune response of scleractinian coral against the challenge of *V. coralliilyticus*, the activities of crucial enzymes in the redox, PO, and apoptosis systems were monitored in *P. damicornis*. Elevated activities were observed for SOD and NOS at 6 h, PO at 12 h, CAT and caspase3 at 24 h in the *Vibrio* challenge group relative to the control group, showing that the redox, PO, and apoptosis systems in this scleractinian coral were activated by *Vibrio* challenge. It also demonstrates that the scleractinian coral could launch full innate immune response against *Vibrio* infection. Substantial ROS and RNS is produced generally by invertebrate hosts to kill the invaded pathogens once recognized (Nyholm and Graf, 2012), the production of ROS or RNS in the coral after challenge was not determined in the present study owing to sample operation difficulty. However, we observed the significant rise of the antioxidase and NOS activities, which might further reflect the production of more ROS and RNS in *P. damicornis* after *Vibrio* challenge. Furthermore, the induced PO would catalyze the production of insoluble melanin to aid in the encapsulation of invaded pathogenic vibrios, and more caspase3 was activated to trigger apoptosis to eliminate injured coral cells containing vibrios and symbionts. Taken together, these results suggest that the infection of *V. coralliilyticus* could activate the redox, PO, and apoptosis systems in *P. damicornis* to initiate the innate immune defense against *Vibrio* infection.

The inductions of these immune components have also been observed in the scleractinian coral under environmental stresses, especially heat stress that causes bleaching (Downs et al., 2002; Mydlarz et al., 2009; Palmer et al., 2011; Tchernov et al., 2011; Yu et al., 2017). This suggests that the triggered immune response in *P. damicornis* by *Vibrio* challenge might be responsible for the decrease in the symbiont density and disruption of the symbiosis between *Symbiodinium* and the coral. On the other hand, coral immunity is crucial to the metabolism and health of the scleractinian coral, because it can regulate and control bacterial community in coral biofilms (Cardenas et al., 2012; Kvennefors et al., 2012), and the bacterial community can provide necessary nitrogen and sulfur resources for the growth and reproduction of the coral (Raina et al., 2009; Lema et al., 2012). Therefore, it was speculated that there was a trade-off between the elimination of a potential pathogen and the maintenance of coral-*Symbiodinium* symbiosis and beneficial bacteria in the scleractinian coral, and the needed balance can be sensitive to environmental changes. This is supported by the observation that the scleractinian coral was more susceptible to bleaching, accompanied by an improved immune response, when it suffered from heat stress (Mydlarz et al., 2009; Palmer et al., 2010; Kimes et al., 2012).

### Repressed Complement System of the Scleractinian Coral at the Transcriptional Level After *Vibrio* Challenge

To understand the molecular mechanisms underlying the response of the coral-*Symbiodinium* association to pathogenic infection, we sequenced the metatranscriptome of *P. damicornis* at 24 h after the challenge of *V. coralliilyticus*. Analysis of differentially expressed genes and corresponding enriched GO terms revealed that significantly upregulated genes were mainly related to the immune response, immune regulation, and cytokine production regulation, and the immune regulation was comprised of the activation of immune response, pattern recognition receptor-activation signal transduction, and positive regulation of immune response. The result was consistent with the increase of immune-related enzyme activities, and agreeable to a previous report on the scleractinian coral challenged by *V. coralliilyticus* YB1 (Vidal-Dupiol et al., 2014). Furthermore, it could be supported by the binding of lectin protein in the scleractinian coral to LPS or Gram-negative bacteria (Kvennefors et al., 2008; Zhou et al., 2017).

However, among the four overrepresented GO terms for the 890 significantly downregulated genes, two terms (*innate immune response* and *defense response*) were also observed in the analysis of the significantly upregulated genes. There were more upregulated genes for immune response, including macrophage mannose receptor, Toll-like receptor, NACHT, LRR, and PYD domains-containing protein and the TNF receptor-associated factor. This indicates that immunity machinery was remolded at the transcriptional level in the scleractinian coral after *Vibrio* challenge. As a result, the genes of complement components such as C3 and CUB were significantly downregulated. The complement system is an important part of the immune system that enhances the ability of phagocytic cells to clear microbes and attack the pathogen’s cell membrane, and multi-components in the complement system had been identified in cnidaria (Kimura et al., 2009). The expression level of complement C3 as the core component has been shown to respond to bacterial challenge in the scleractinian coral *Acropora millepora* (Brown et al., 2013). This demonstrates that the immune ability of the complement system was suppressed in *P. damicornis* after *Vibrio* challenge. These results together suggest that the *V. coralliilyticus* infection can reconfigure the immune landscape, triggering the innate immune response (including the redox, PO, and apoptosis systems), but repressing the response of the complement system in *P. damicornis*.

### Disordered Metabolism of the Symbionts During Host Immune Response

To have a deeper insight into the effect of *Vibrio* challenge on the coral-*Symbiodinium* symbiosis, we further examined the functions of differentially expressed genes in the symbionts. We only identified 10 significantly upregulated genes, which were mainly related with nucleic acid and polysaccharide metabolisms. Among the 133 downregulated genes identified, major enriched GO terms were relevant to amino acid catabolism. These altered metabolic pathways were all essential for the maintenance of the symbiont and its symbiosis with the coral host. Similar metabolic alteration of the symbionts has been reported in the other scleractinian corals under heat stress and depth change, which was accompanied by the shift of *Symbiodinium* genotype (Cooper et al., 2011; Jones and Berkelmans, 2011). The metabolic changes in the present study might have resulted from the interaction of the two symbiotic partners in response to *Vibrio* infection. The scleractinian coral needed more organic nutrition from the symbiont to meet the massive demand of immune energy after *Vibrio* challenge, and the symbiont could not photosynthesize at full capacity because of the excessive oxidative stress and chlorophyll content decline. The odds between the scleractinian coral and *Symbiodinium* might eventually lead to the collapse of the symbiosis.

## Data Availability

The datasets generated for this study can be found in SRA, SRP133521.

## Ethics Statement

All animal-involving experiments of this study were approved by the Ethics Committee of Hainan University and local government.

## Author Contributions

SZ and ZZ conceived and designed the experiments. SZ, JT, YW, and ZZ performed the experiments. SZ, ZL, and ZZ analyzed the data. JT and YW contributed reagents, materials, and analysis tools. SL and ZZ contributed to the discussion and wrote the manuscript. All the authors read and approved the final manuscript.

## Conflict of Interest Statement

The authors declare that the research was conducted in the absence of any commercial or financial relationships that could be construed as a potential conflict of interest.

## Footnotes

1 http://hannonlab.cshl.edu/fastx_toolkit/index.html
